# Evaluation of Confusion Behaviors in SEI Models

**DOI:** 10.3390/s25134006

**Published:** 2025-06-27

**Authors:** Brennan Olds, Ethan Maas, Alan J. Michaels

**Affiliations:** Virginia Tech National Security Institute, Blacksburg, VA 24060, USA; beolds@vt.edu (B.O.);

**Keywords:** Radio Frequency Machine Learning (RFML), Specific Emitter Identification (SEI), RF Fingerprinting, confusion matrices

## Abstract

Radio Frequency Machine Learning (RFML) has in recent years become a popular method for performing a variety of classification tasks on received signals. Among these tasks is Specific Emitter Identification (SEI), which seeks to associate a received signal with the physical emitter that transmitted it. Many different model architectures, including individual classifiers and ensemble methods, have proven their capabilities for producing high accuracy classification results when performing SEI. Though the works studying different model architectures report on successes, there is a notable absence regarding the examination of systemic failures and negative traits associated with learned behaviors. This work studies those failure patterns for a 64-radio SEI classification problem by isolating common patterns in incorrect classification results across multiple model architectures and two distinct control variables: Signal-to-Noise Ratio (SNR) and the quantity of training data utilized. This work finds that many of the RFML-based models devolve to selecting from amongst a small subset of classes (≈10% of classes) as SNRs decrease and that observed errors are reasonably consistent across different SEI models and architectures. Moreover, our results validate the expectation that ensemble models are generally less brittle, particularly at a low SNR, yet they appear not to be the highest-performing option at a high SNR.

## 1. Introduction

Radio Frequency Fingerprinting (RFF), often termed Specific Emitter Identification (SEI), is the process of associating a received signal with an emitter based on features present within a transmission [1]. These radio frequency genuine features are able to be used for device identification as they are unique to each specific emitter, being caused by intrinsic hardware discrepancies created during manufacturing processes [2]. In recent years, machine learning approaches have been implemented to isolate these features and increase SEI performance while eliminating the need for human-defined expert features [3,4,5,6,7,8,9,10,11], which require extensive time and effort when compared to the feature extraction capabilities of machine learning (ML) models.

A variety of different ML architectures have been implemented in the creation of SEI models, including Convolutional Neural Networks (CNNs) [3,4], Deep Neural Networks (DNNs) [5], Recurrent Neural Network (RNN) approaches such as Long Short-Term Memory Networks (LSTMs) [6], decision trees [7], ensemble methods such as Adaboost and Random Forest [8], and other network types [9,10]. Each of these architectures, when trained on data collected from real-world emitters, has produced successful classification results when applied to data collected in a variety of conditions. Of these conditions, two in the literature that produce large variations in model accuracy are varying signal-to-noise ratios (SNRs) [6,7,11], which produces a degradation in model performance as the noise present in a collected signal increases, and varying the amount of data used in the training of a model, which shows that the more training data used, the stronger a model’s performance [10].

SNR quantifies the magnitude of a desired signal versus noise in a transmission [12]. A higher SNR value indicates lower relative noise present when compared to the desired signal, while a lower SNR value indicates the opposite. SNR is a common consideration when examining the strength and applicability of SEI models to real world data, as noise levels in a captured signal can vary greatly. This variation in noise can impact a model’s ability to isolate the radio frequency genuine features in a signal [13], which are required to perform proper classification and reduce identification accuracy [14]. SEI models that are able to isolate these features at both high and low SNR values are therefore highly desired to create robust classifiers. Most SEI literature tends to focus on the performance of signals collected at extremely high SNRs (i.e., >20 dB $\frac{E_{s}}{N_{0}}$) and tends to ignore the effective decision statistic gain that is derived from a neural net being presented with substantially more than one symbol at a time [15]. In practice, this leads to over-inflated SEI classification results. The common trend found in the literature studying SEI performance over SNR shows that as SNR decreases, so does model performance [6,7,11].

One attempt in the literature to overcome the degradation of model performance across SNRs is to use fusion models, or ensemble methods. Ensemble methods train multiple weak learners and apply the knowledge learned by each to the overall classification decision [16]. The main ensemble learning approaches for SEI apply bagging techniques [17], which train multiple weaker learners in parallel and combine their classification decisions and boosting techniques [8,18], which train multiple weaker learners in succession and pass the learned features of each learner to the next to inform the overall decision. When compared to the classification accuracy results of one singular learner, ensemble methods show an increase in accuracy at lower SNRs [18] compared to singular models.

Though existing works examine how SNR and training data quantities impact the classification accuracies of trained models, these approaches focus on maximizing classification accuracy, while failing to analyze the trajectory of improper classification results. Classification accuracy is reported as such; however, no consideration is given to the patterns with which the models misidentify emitters as a variable of interest changes. A better understanding of how emitters are commonly misidentified and what failure patterns different model architectures produce can help to deepen the comprehension of RFML features learned within SEI algorithms and strengthen their robustness in deployment.

This work details a novel approach to determine the failure patterns of various SEI algorithms by evaluating how each algorithm degrades across two variables: SNR variations and the quantity of data used to train an architecture. Four different model architectures were selected for testing in this approach, each chosen for their prior success in performing SEI in the literature as well as the intentional disparity between model architectures. Disparate architectures were selected in order to determine if model degradation patterns were unique to any specific type of algorithm or a common consideration for SEI as a whole. Further, a novel ensemble architecture was designed for this work to produce a trained model that produced consistent classification accuracies regardless of variations in SNR and error behaviors that showed no clear pattern. Additionally, this work evaluated SEI algorithms at much higher dimensionality (64 radios/classes) than most currently available.

This paper is organized as follows: Section 2 describes how a large, real-world dataset was generated for use in this experiment, as well as how these datasets were segmented into varying SNR and training dataset sizes. Section 3 details the various model architectures selected for use in this experiment and the reasoning for the selection of each. Section 3 defines how each model’s results were evaluated to isolate failure patterns and describes the expected results of studying multiple architectures. Section 4 compares and contrasts patterns observed in each of the models using a novel, three-dimensional representation of the confusion matrices and discusses the trade-offs of using the designed ensemble model. Finally, Section 5 discusses conclusions and future work that could improve upon this work to build more robust RFML-based SEI algorithms.

## 2. Dataset Generation

Machine learning architectures for SEI require large amounts of data per class to train a model that can accurately differentiate between emitters. Synthetic data is often used for model design, as it can be quickly and efficiently generated. Real-world data, however, produces models that are able to better perform when faced with unpredictable behaviors that arise in deployed use cases. For SEI algorithms in particular, which largely learn the hardware variances or nonlinearities of a group of otherwise equivalent emitters, a live over-the-air dataset is crucial [19]. In this experiment, a real-world dataset was generated in order to train model architectures that maximized performance in deployed usage environments.

### 2.1. Hardware

The dataset created for this work, denoted $\Omega_{64}$, was collected from 64 different emitters. In order to collect data from multiple devices in an efficient manner, the Blind User-Reconfigurable Platform, otherwise known as the BURP machine [20], was used as the emitter host. This machine was selected for use in dataset generation as it was designed to create large, real-world RF datasets. This machine can support up to 120 USB-based software-defined radios, each made to transmit one at a time across a channel randomly selected among those available. This random selection allows for biases in a dataset caused by the unbalanced presence of one device’s radio frequency genuine features, or channel variations caused by the over-utilization of a specific channel used for transmission, to be mitigated. Further, the datasets produced by the BURP machine are real-world labeled datasets.

**Figure 1:**
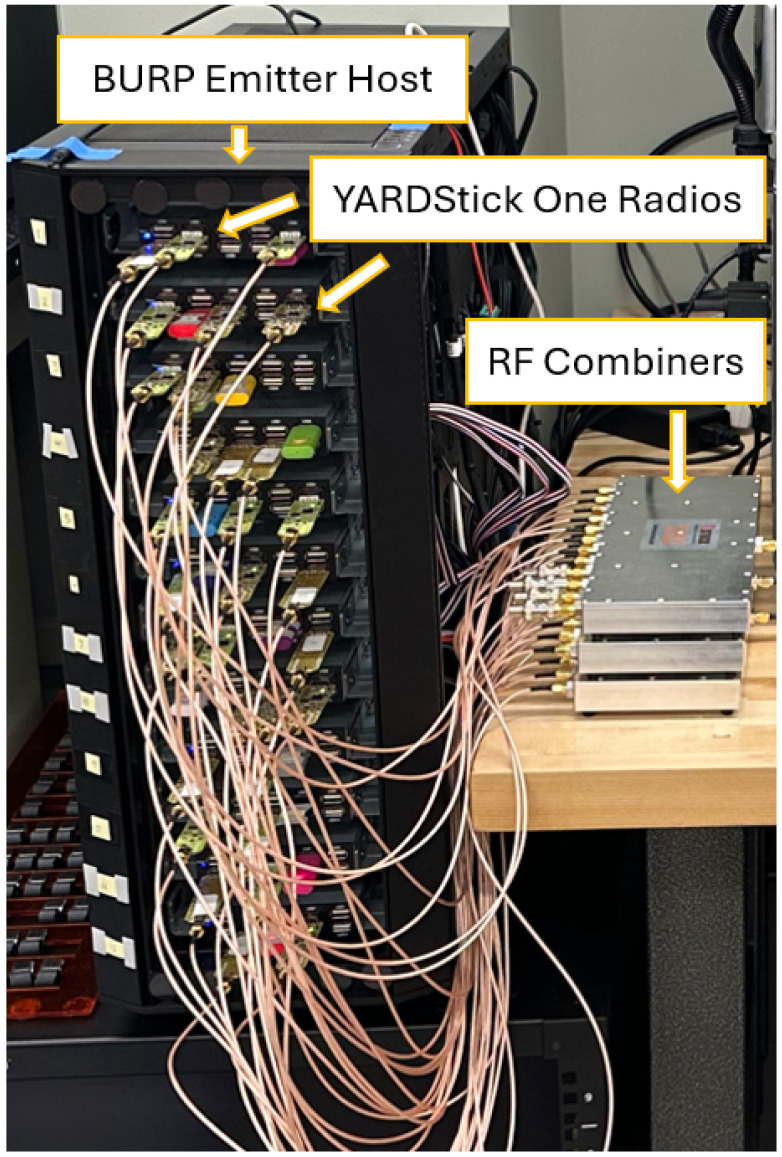
A image of the BURP machine with YardStick One Radios connected to RF combiners.

The 64 emitters selected for use in this experiment were YARD Stick One test tools [21] due to their prior success in generating high-quality data with the BURP machine [20,22,23] as well as their low unit cost. Each burst transmitted by BURP selects an individual emitter to transmit a random string of any specified byte length at any modulation scheme permitted by emitter hardware. In this experiment, the modulation schemes used were OOK, MSK, 4-FSK, 2-FSK, and GFSK, as defined by the YARDStick One’s hardware capabilities [21]. A data rate of 31250 baud and center frequency of 416.4 MHz were utilized for each transmission. In order to minimize the presence of pathloss variations and other SEI performance biases derived from channel variations caused by antenna location changes, which have been shown to be a variable that impacts model performance [13,14], all emitters were connected to a single antenna through RF combiners, as shown in Figure 1. This antenna was then positioned in a fixed location to mitigate potential channel variations caused by antenna movement. Further, the use of a consistent antenna allows for the study of the impacts of applying SEI algorithms to the entire hardware chain of an emitting device. In the literature, emitter identification problems are often correlated with only the antenna hardware [24]; however, performing SEI on data collected using a static antenna shows equivalent performance to varying antennas [25], thus warranting the consideration of the radio, cables, and other connected hardware. Due to the antenna hardware being held constant, the term emitter identification may not be sufficiently relevant for defining the classification tasks that the generated datasets are being used for; therefore, these studies fall in the RF Fingerprinting problem space.

**Figure 2:**
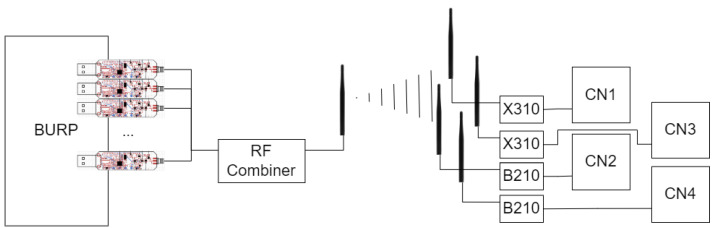
A block diagram of the experimental setup utilized to create the dataset used in this work.

Four different receivers were used in this experiment for collecting the data, each receiving data from the current emitter at the same time through their own antennas, located in close proximity. Two USRP B210 and two USRP X310 software defined radios (SDRs) were selected as receivers and placed in a direct line of sight with the emitter at approximately 3 m. The close proximity of the emitter to the receiver was selected to ensure that collected data had a high SNR, which allowed for the strength and clarity of the baseline received signals to be as strong as possible. To further improve SNR, the experiment was performed in a controlled lab environment. To minimize interference from external emitters and other noise present in the ISM bands, the radios were made to operate in isolation, separated from any other potentially emitting devices during experimentation. Further, the design of the BURP machine created variations in the channels used to transmit signals, allowing for noise present in one channel to not impact an entire dataset [20]. Each SDR was connected to a custom designed collection node (CN1-4) designed for use with the BURP machine to allow for the storage of large RF datasets. Each collection node housed a 14 TB hard drive disk specifically for RF capture storage, allowing for the large transmissions used in this experiment to be collected all at once. To train and evaluate SEI models with the collected data, four NVIDIA Tesla V100-PCIe GPUs were used. A block diagram detailing the data collection setup is shown in Figure 2.

### 2.2. Dataset Contents

The dataset, $\Omega_{64}$, was generated to examine the failure patterns created by varying SNR and the quantity of data used for training. This dataset contained transmissions of random strings of 1024 bytes, which was a byte length selected to represent realistic transmissions that may be captured in a usage scenario. It was found that each burst of length 1024, when processed for use in machine learning algorithms, produced approximately 244 examples for training a network. In this work, an example represented a subsection of 128 different successive complex I/Q pairs taken from a burst at 250 Ksps sample rate.

In order to allow for the variation of the quantity of data containing radio frequency genuine features used for model training, large amounts of raw data were collected and processed. A minimum of 720 bursts of length 1024 bytes were transmitted per emitter, resulting in approximately 175,000 examples per SEI class. This value was selected based on the work of [10], which found that model accuracy increases at a linear rate of up to $10^{5}$ data points per class. After $10^{5}$ data points per class, the increase in performance gains continues, but the rate of growth begins to diminish. Additionally, at $10^{4}$ data points per class, an accuracy greater than 50% in [10] was achieved, which is deemed acceptable for SEI. However, it is worth noting that the dimensionality of SEI explored in that prior work was much lower than in the current work, leading to an anticipated increase in the amount of data collected. In total, 175,000 examples per class were selected to ensure that a range of training data quantities could be utilized that each produced models with varying accuracies, and further ensured that additional data was transmitted so that the desired quantities could be captured while accounting for potential unusable bursts produced by the BURP machine, as defined by [20].

After the raw data, collected in SigMF format, was processed into examples usable for training a network, the resulting datasets were broken into training, validation, and test subsets using an 80%, 10%, and 10% split. This resulted in the dataset containing $8.96\times 10^{6}$ training examples and $2.24\times 10^{6}$ validation and test examples. The resulting 80% training subsets could be broken down further to examine the effectiveness of training SEI models on various amounts of input data. The subsets used were 1562 examples, 15,625 examples, and 78,125 examples per class. As previously stated, it was found that performance benefits increase approximately linearly as the volume of examples used for model training increase up to $10^{5}$ data points per class, with an acceptable accuracy being reached in the neighborhood of $10^{4}$ data points per class. A division of 1562, 15,625, and 78,125 examples per class allowed for each of the architectures used in this experiment to be tested with a data quantity below, equal to, and greater than that expected to achieve acceptable SEI performance.

### 2.3. SNR Variation

In order to vary the SNR present in the collected signals, synthetic noise was added to test datasets before being inserted into trained models. The SNR of each individual example from the test dataset was determined before being fed to the trained network. To calculate signal power, the following equation was used:

$$signal\;power=10log_{10}\left(\sum_{i=1}^{n}real_{n}^{2}+imag_{n}^{2}\right)$$

The raw input was split into real and imaginary components and the current signal power was calculated by taking the mean of the sum of the squares of the real and imaginary components across the signal. The resulting output was converted to dB, and the difference between signal power and the desired SNR was used to determine the necessary amount of additive noise required.

$$noise\;power={\left(\left(10^{\frac{signal\;power-SNR}{10}}\right)/2\right)}^{0.5}$$

SNR was used in this work to categorize datasets at the signal level; however, it is also a necessary component of the decision statistic. SNR differs from accuracy in that it is a question of the CNN that is run against the overall frame (decision statistic) an, thus, the use of SNR, particularly when measured over a span of time, seeks to help normalize those figures.

Generated Random Gaussian White Noise (RGWN) in Python 3.12 was used to adjust the individual example’s SNR to any desired value. Each time noise was added, the initial condition was randomized, ensuring that the noise added to each example of the signal was different and therefore would not cause the models to learn features related to noise rather than the desired signal.

In this experiment, SNR values between −10 dB and 40 dB were selected for examination, extending the lower end of SNR ranges considered within the literature [6,7,11]. The datasets generated are denoted as $\Omega_{64-SNR}$, where SNR is replaced with the numeric value representing the ratio, for example, the training dataset with an SNR of 20 dB is denoted as $\Omega_{64-20}$.

### 2.4. Comparison to SEI Literature

PARADIS is an example of an SEI technique achieving extremely high accuracy in identifying 802.11 Network Interface Cards (NICs) with varying levels of SNR [20,26]. The YARDStick emitters produce real world data that is better analyzed with an SEI model [27,28]. This is because targeting hardware imperfections produce higher accuracy results [3,29,30]. What separates the YARDStick experimental setup from other SEI models analyzing real world data is that the quantity of devices allowed us to produce 64 classes of data and multiple models to be trained and compared. Other works trained SEI models on the transient state of a signal; this SEI technique allows models to train on the portion of the signal where hardware imperfections are the most apparent in signal output [4]. However, this attempt at improving SEI is uncommon, as isolating the transient of a signal is difficult [31,32]. Applying SEI to data in the physical space is not restricted to emitter hardware. These techniques are also applicable to training models on basic radar parameters such as time of arrival, angle of arrival, or pulse repetition interval [33].

A different strategy previously employed to improve SEI model performance is manipulating CNN architecture [34]. The previous literature describes adding convolutional and rectified Linear Unit (reLU) layers, yielding greater performance [35]. This approach is similar to what ensemble learning represents, allowing for greater complexity and deeper learning from models proven to succeed with SEI, improving the performance capabilities of SEI models [4,30,35]. While previous work might have sought to improve a particular model, ensemble methods allow for multiple models to learn and be compared to one another.

Other experimental setups have obtained datasets by conditioning the output of a signal [31]. This strategy seeks to exploit the high quality data seen in the hardware imperfections of emitter devices [29,36,37]. This study took a similar approach by training models with real data at various SNRs, comparing accuracy results. However, while other works simply illustrate a higher performance over SNR, this study’s experimental setup was more focused on identifying biases that different SEI models might have towards imperfections when making a prediction [38,39]. Previous work also used a smaller range of SNRs, fewer transitting devices, and synthetic data [40].

## 3. ML Models

### 3.1. Model Architectures

Five different model architectures were trained and evaluated to examine RFML failure patterns. These model architectures were selected based on their existing successes when implemented for RFML classification tasks in the literature, as well as the disparity of their structures. Examining models with various different structures allows for conclusions to be made regarding patterns and weaknesses in SEI algorithms as a class, instead of applying them to only one type of architecture. The model architectures used in this work included a Convolutional Neural Network (CNN), Fully Convolutional Network (FullConv), Long Short-Term Memory (LSTM) Network, Convolutional Long Short-Term Memory Deep Neural Network (CLDNN), and an ensemble method designed to combine the strengths of the prior models. The ensemble model trained each of the prior four architectures in parallel on the same inputs to create the set of weak learners. Once trained, the model states were aggregated using a final CNN layer to produce the classification decision. A summary of the architectures can be found in Table 1.

### 3.2. Model Evaluation

**Figure 3:**
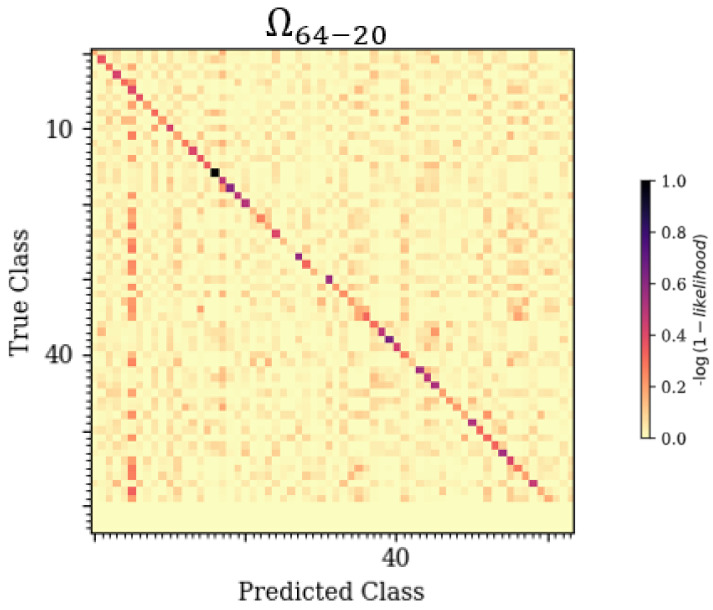
A 2-D confusion matrix relating True-Class to Predicted-Class for the trained CLDNN model tested at an SNR of 20 dB (dataset $\Omega_{64-20}$).

To determine the patterns with which each of the model architectures incorrectly classified emitters, a novel technique was implemented which utilized stacked confusion matrices and their cross-sections to create a 3-D representation of classification error patterns across a variable of interest. For each trained model, this technique stacked the confusion matrices across all applicable test datasets. A confusion matrix is a 2-D graphical representation correlating Predicted-Class to True-Class. An example confusion matrix can be seen in Section 4.2, Figure 3, displaying the classification results from applying dataset $\Omega_{64-20}$ to the trained CLDNN model.

Individual confusion matrices could be created for each trained model over all test datasets and stacked to create a 3-D representation of confusion matrices showing Predicted-Class on the X-axis, True-Class on the Y-axis, and the variable of interest on the Z-axis (e.g., SNR or data quantity). Common classification patterns across the Z-axis could then be seen, appearing as “veins” down the 3-D shape. These “veins” could be further examined by taking cross-sections of the 3-D depiction across different dimensions to isolate error patterns of a specific emitter or variable value.

When examining the 3-D confusion matrix representation, the desired behavior was that the only recurrent pattern in the Z-dimension would occur on the diagonal matching True-Class to Predicted-Class. This meant that accurate classifications were consistent and implied that inaccurate classifications were random. It was expected, however, that certain emitters would be commonly classified as another specific emitter across the Z-axis due to some closeness in the learned feature space. This recurring incorrect classification would appear as a “vein” down the Z-axis off of the diagonal, indicating a misclassification pattern.

When examining a cross-section of the 3-D shape taken from the X and Z dimensions, or from the Y and Z dimensions, it was expected that certain X or Y classes would show across most Z values, indicating a pattern in incorrect classification. Further, these cross-sections could be utilized to obtain a graph of the frequency at which each emitter would be classified as a different emitter across the Z-axis. This allowed for the most frequent misclassifications to be determined and isolated. Additionally, the derivative taken along the free parameter of interest (e.g., SNR) gave insight into the rate of change of classification accuracies as a variable of interest changed; this allowed for one to quickly and clearly determine if a specific radio was more prone to misclassification as a variable of interest changed. As such, we explored the shifts in joint probability statistical distributions to attempt to discern consistent patterns in incorrect classifications, which we could potentially to infer as minimum distances between radios within the learned feature space.

Across various model architectures, it was expected that patterns in emitter misclassification would be consistent across similar architectures but different in more disparate architectures. This was due to the fact that SEI was performed based on specific signal characteristics. Due to this, if two emitters were commonly misidentified as one another by multiple architectures, it could be assumed that those architectures were deriving similar features drawn from the received signals for classification.

## 4. Results and Analysis

The results are split into four subsections: baseline performance, which indicated the accuracies of the trained models across both of the variables of interest (i.e., SNR and data quantity); failure analysis, which indicated the failure patterns found from analyzing model performance across SNR and varying training data quantities; ensemble analysis, which indicated the trade-offs of utilizing an ensemble method to mitigate the failure patterns of individually weaker models; and entropy analysis, which provides a qualitative metric for error behavior.

### 4.1. Baseline Performance

**Figure 4:**
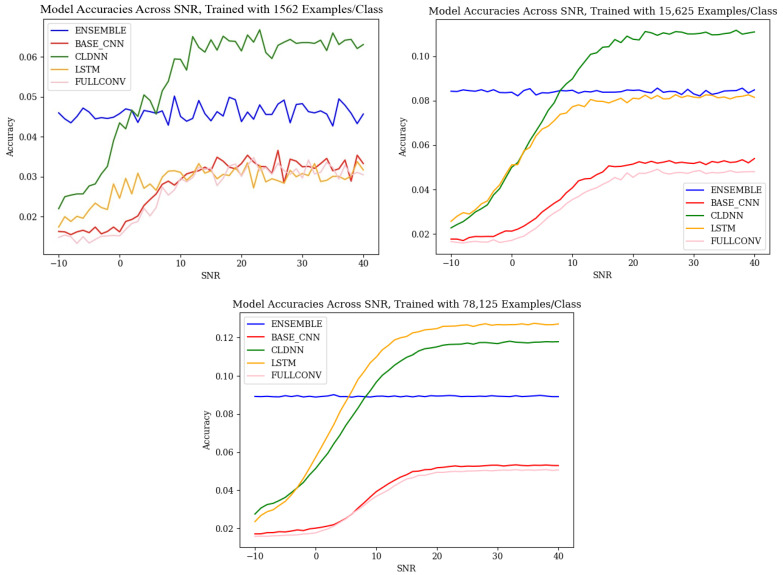
Baseline accuracies across SNR for the model architectures used in this work, trained with 1562 ex/class, 15,625 ex/class, and 78,125 ex/class.

The baseline performance of each of the model architectures, shown in Figure 4, aligned with the common trends discussed in the literature. As SNR increased, the accuracies of the CNN, CLDNN, LSTM, and FULLCONV architectures increased. At an SNR of approximately 10–20 dB, the accuracies of these architectures began to plateau, which implied that above this threshold, the amount of noise present in the signal was not strong enough to impact the radio frequency genuine features being learned by each architecture. The baseline accuracies of the ensemble models did not follow the same trend. Regardless of SNR, ensemble accuracy remained fairly constant, implying that the ensemble architecture designed for this work was not significantly impacted by variances in SNR. For completeness, the effective number of data symbols contained in each input example was 1024, so the effective decision variable SNR was approximately 21.1 dB higher than that shown as the SNR measured as $\frac{E_{C}}{N_{0}}$.

When observing the accuracies of models trained using different quantities of input training data, it can be seen that the results followed the expected trends, as defined by [10]. As the quantity of training data increased, the classification accuracies of each model architecture increased as well. An increase from 1562 ex/class to 15,625 ex/class produced the most notable increase in accuracies, and an increase from 15,625 ex/class to 78,125 ex/class produced an increase in performance, although much less significant. It is worth noting that at low SNR values, training data quantity did not greatly impact classification accuracy. The performance benefits of utilizing larger amounts of training data were best realized above SNR values of 0 dB. This relationship, however, did not hold true for the ensemble model designed for this work, as ensemble classification accuracies remained consistent across all SNR values. For the ensemble architecture, regardless of SNR, an increase in training data quantity was seen to produce an increase in classification accuracy.

While the accuracies of these models, ranging from a worst case scenario of 1% accurate to a best case scenario of 12% accurate, were relatively low compared to the accuracies of other SEI models in the literature, it is important to consider that these models were designed and tested originally on synthetic data, and, therefore, performance was expected to decrease when applied to real-world data. Though the accuracies were low, the trained models at each SNR still outperformed random guessing, which, for a classification problem with 64 classes, has an accuracy of 1/64 or 1.56%. Further, this lower classification accuracy helped to highlight error patterns across variables of interest as classification mistakes were more common.

**Figure 5:**
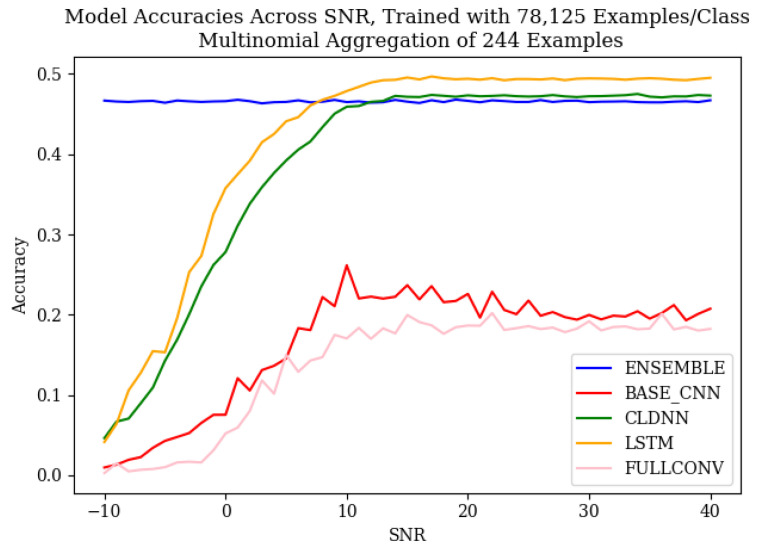
Baseline accuracies across SNR for the model architectures used in this work, trained with 78,125 examples/class, after the multinomial decision aggregation technique was applied to a 1 s signal capture.

To mitigate these low results, the multinomial-based decision aggregation technique defined in [15] was utilized. When applied to the models using the equivalent of one second of observation data (244 successive examples), the accuracy of each model increased significantly, as can be seen in Figure 5, resulting in even the worst model’s accuracy after the application of the multinomial being greater than the best accuracy before the multinomial at higher SNR values. The multinomial method of [15] yielded the greatest advantage when classification errors were homogeneous, leading to the substantial gains in the ensemble model.

### 4.2. Failure Analysis

**Figure 6:**
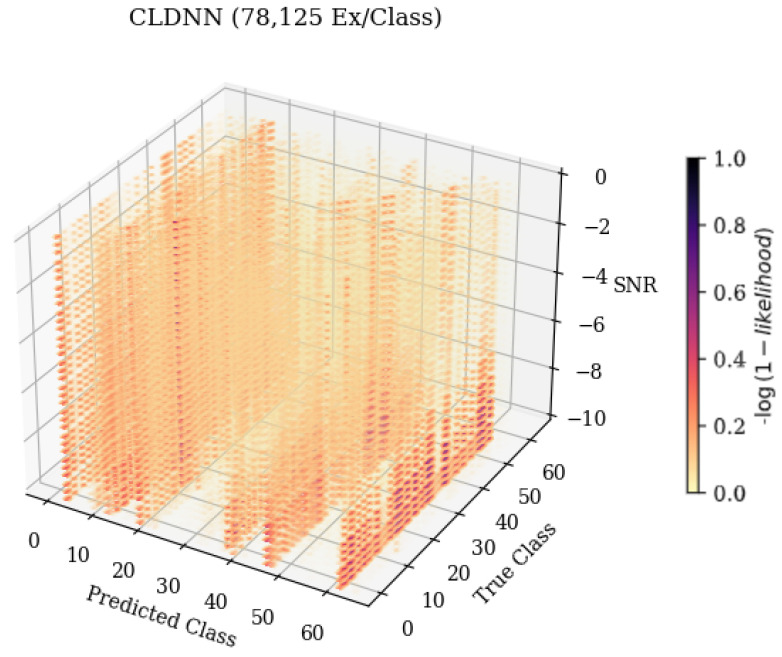
An example 3-D confusion matrix showcasing how error patterns changed across a variable of interest. As the variable on the Z-axis, SNR, changed, the frequency that the model classified emitters also changed, with more frequent classifications being depicted in darker colors.

A novel 3-D confusion matrix modeling technique was developed for this work in order to examine the failure patterns of model architectures across a variable of interest. An example image showcasing a zoomed-in subset taken from a trained CLDNN model’s 3-D matrix can be seen in Figure 6. This image stacked confusion matrices, as shown in Figure 3, across a variable of interest to illustrate recurring error patterns across the Z-axis. More frequent classifications appear in darker colors, providing improved visualization for isolating and identifying patterns where errors occurred. A deeper understanding of these patterns allowed for improvement upon the trained behaviors. Most prior work only considered small numbers of SEI emitters, motivating the need for better visualizations for high-dimensionality SEI problems.

**Figure 7:**
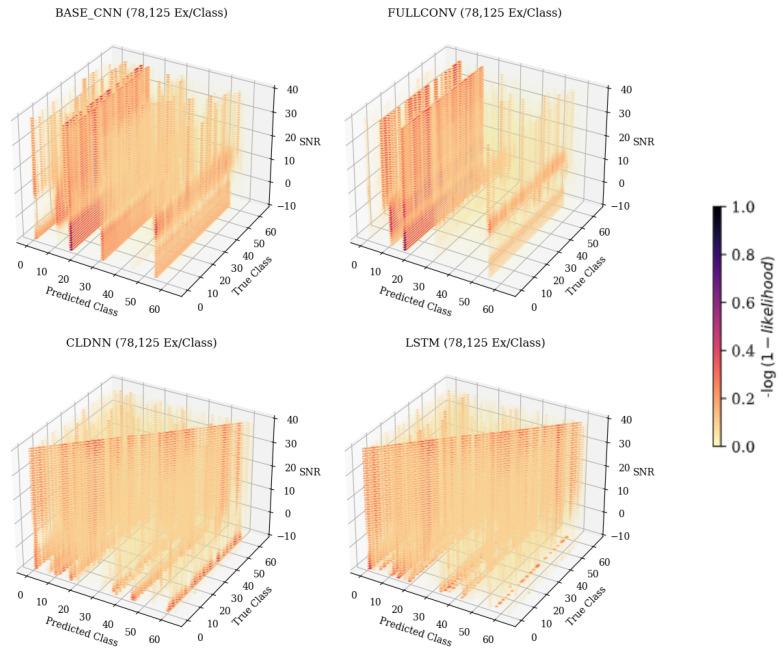
The 3-D confusion matrices depicting accuracies across SNR for each of the individual model architectures. Models depicted were trained with 78,125 ex/class. The darker the color, the more common the classification.

**Figure 8:**
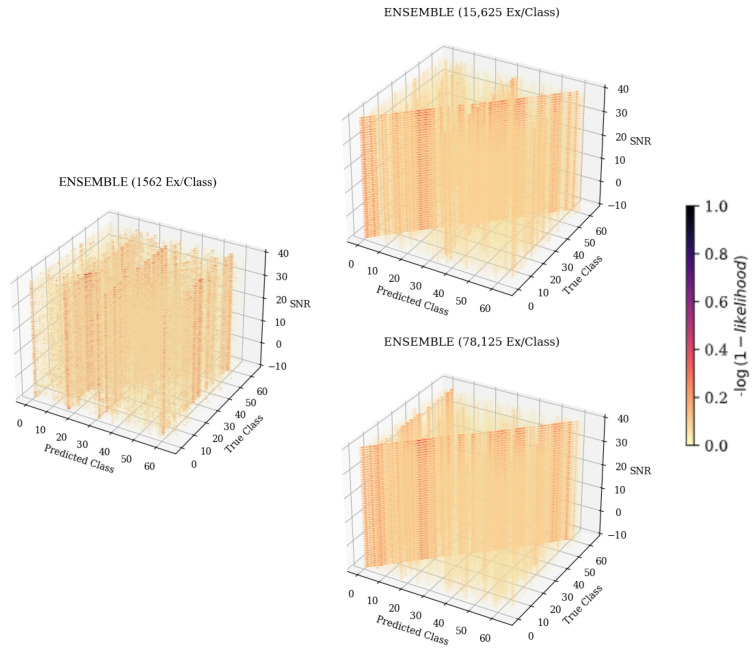
Three-dimensional confusion matrices depicting accuracies across SNR for the ensemble architecture. Models were trained with 1562 ex/class, 15,625 ex/class, and 78,125 ex/class.

When examining Figure 6, predicted class 61, it can be seen that as the SNR increased, the frequency at which the emitter was classified as 61 decreased. The resulting 3-D matrices created from this technique for the CNN, CLDNN, FULLCONV, and LSTM architectures trained with 78,125 examples per class can be seen in Figure 7 for data collected from CN1 for 78,125 examples per class. Additionally, the resulting matrices for the ensemble model designed for this work can be seen in Figure 8.

#### 4.2.1. Failure Analysis: Data Quantity

When examining the 3-D confusion matrices, the desired result was that the only recurrent pattern would show on the diagonal matching True Class to Predicted Class, indicating that emitters were repeatedly classified accurately and implying that mistakes were random. The expected result, however, was that error patterns would appear off of the diagonal, indicating that specific emitters were consistently misclassified across a variable of interest. Similar architectures were expected to learn similar signal features and therefore were expected to develop comparable mis-identification patterns. More disparate models that were expected to perform classification based on different learned features were not expected to produce the same patterns.

An analysis of the 3-D confusion matrices across training data quantities in Figure 7 shows that patterns other than the desired diagonal arose regardless of SNR values and model architecture. CNN, FULLCONV, and LSTM models trained using 1562 ex/class did not show the desired diagonal pattern at any SNR value, and models trained using larger quantities of training data showed recurring patterns across SNRs off the diagonal, indicating that classification errors were not homogeneously distributed.

**Figure 9:**
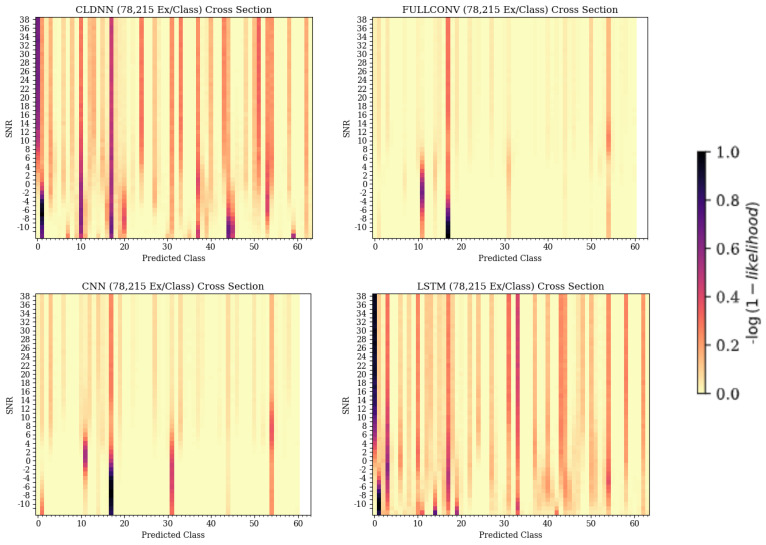
Cross-sections taken from each model’s 3-D confusion matrix depiction, showing Predicted Class on the X-axis and SNR on the Y-axis. The dark colors signify the emitters that the models were consistently predicting when the true emitter was emitter 0.

In the CNN, FULLCONV, and LSTM models trained with 1562 ex/class, recurring patterns commonly incorrectly predicting a specific emitter could be seen regardless of the true emitter, and the desired diagonal was not present. Parallel patterns down the Z-axis and across a predicted class, regardless of true class, could be seen, indicating that the trained models were identifying most bursts as a small subset of possible emitters. Taking cross-sections showing Predicted Class on the X-axis and SNR on the Y-axis further emphasized this pattern, allowing the specific emitters in this smaller subset to be determined for each model type. These cross-sections for the CNN, CLDNN, FULLCONV, and LSTM models trained with 78,125 Ex/Class can be seen in Figure 9. Note that for the CLDNN and LSTM models, which had higher classification accuracies than the CNN and FULLCONV models, there were a significantly higher amount of classes being frequently predicted. This indicated that errors of the CLDNN and LSTM showed less repeated error behaviors than that of the CNN and FULLCONV, as the models were commonly predicting from a larger subset of emitters. An ideal cross-section would show a vertical line located at the Predicted Class matching the True Class from which the cross section was taken (i.e., 0 for True Class 0), with no other classifications. As these models were not able to be 100% accurate, the more distributed the predicted classes, the less frequent the classification errors were for the True Class.

When examining each of the trained models, it can be noted that the emitters being most commonly predicted were the same across each architecture, being radios 11, 17, and 54. Emitters all being classified into this smaller subset could indicate that the model was attempting to maximize performance by always guessing from the specific emitters, which resulted in a guessing strategy that was more effective than random guessing and more efficient than learning features. The subsets matching across model architectures, however, indicated that this was not the case. The recurring classification as an emitter from a smaller, specific subset, regardless of true emitter and architecture, implied that the features learned by these architectures from these emitters generalized more to the whole dataset, causing the specific features of emitters outside of the subset to be indistinguishable. These emitters shared some similarity in the learned feature space(s) that was biased towards errors in these classes. Despite this finding, these emitters were treated no differently than any other node in the experiment. The goal of this study was to isolate error behaviors within high dimensionality datasets; the cause of these behaviors will be explored in future work.

The CNN and FULLCONV models trained using larger quantities of data per class began to produce the desired diagonal pattern indicative of proper classification; however, the common classification error patterns found in the lower training data quantity models were still present. For specific predicted classes, these error patterns could occur at higher SNR values, lower SNR values, or across all SNR values. The presence of error patterns across SNR ranges implied that the signal features learned by the models were the cause of incorrect classification patterns rather than features learned from the noise present within a signal.

At all training data quantities, the CLDNN model outperformed the CNN and FULLCONV models in both accuracy and the presence of the desired classification patterns. At the first two data training quantities, the CLDNN outperformed the LSTM; however, at the highest quantity, the LSTM outperformed the CLDNN. At the lowest data quantity, the desired diagonal could be seen along with other incorrect classification patterns across all SNR values of the trained CLDNN. In CLDNN models of all training data quantities, when observing patterns off the diagonal, the veins appearing down the Z-axis indicated a much more random pattern of misclassification across SNRs when compared to the other models. The same patterns that appeared for the other model architectures could still be noticed, however, further supporting that the error patterns were influenced by specific emitters in the dataset rather than being feature- or model-dependent.

The recurring model weaknesses of generalizing classifications to a smaller subset of emitters and misclassification patterns being emitter-dependent rather than feature- or architecture-dependent result in SEI algorithms that were easily exploitable by potential attackers. Model generalization to a smaller subset can be used by attackers who are aware of this weakness, allowing them to transmit on an emitter known to commonly be misidentified as one from the known predicted subset and therefore go unidentified. SNR-independent misidentification creates a more forgiving environment for attackers, allowing them to be potentially misidentified even when a malicious signal is captured with as little noise as possible. Finally, emitter-dependent rather than model-dependent errors indicate a weakness in SEI algorithms as a whole, as the features being learned by differing implemented architectures result in models that suffer from the same weaknesses. These radio-dependent error patterns can be used by attackers, regardless of architecture, in a similar manner as generalization to a smaller subset of emitters, allowing attackers to transmit on a radio known to confuse models and therefore remain unidentified.

#### 4.2.2. Failure Analysis: Rates of Change in Mis-Classifications

**Figure 10:**
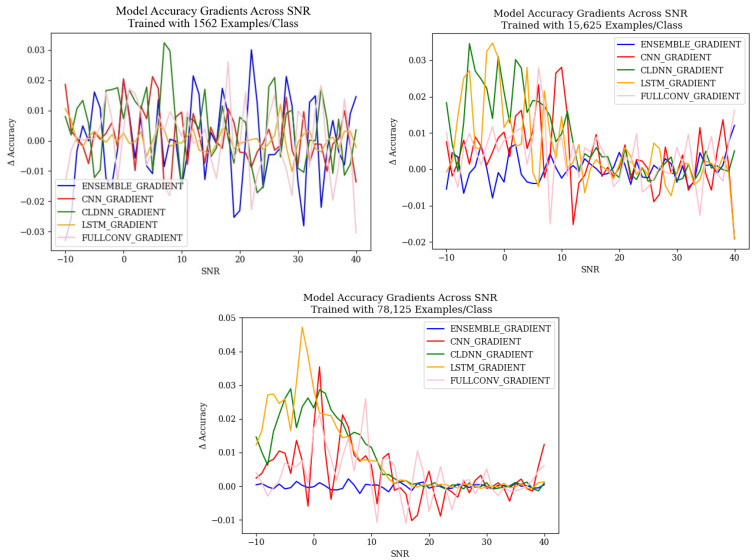
Model accuracy rate of change for each of the explored models.

To further understand the confusion behaviors that result in classification errors due to the SNR, the rate of change of model accuracy results across SNRs was examined. Figure 10 shows the rate of change of each model architecture’s overall accuracy as the SNR changed, trained with each training data quantity. The resulting graphs for the models trained with 15,625 and 78,125 examples/class shows the expected behavior. As SNR increased, the rate of change of accuracy results increased until around 0 dB and then decreased until it began to fluctuate around 0 at approximately 20 dB SNR. This indicated that as SNR increased to 0 dB, accuracy increased at a rapid rate. After 0 dB, accuracy continued to increase, although not as rapidly, and after 20 dB, the accuracy reached a maximum steady value. This pattern differed for the ensemble architecture, as the rate of change fluctuated around 0 for all SNR values, indicating that the accuracy of the ensemble model was fairly constant. The presence of this pattern in models trained using 15,625 examples/class indicated that models trained with over 10,000 examples per class were able to reach a consistent accuracy result at higher SNR values, validating the results of [10]. For the models trained with 1562 examples per class, it can be seen that the rate of change of each model’s accuracy roughly followed the same trend, although results generally showed much more drastic fluctuations. This implied that error patterns of models trained with 1562 examples per class were still impacted by SNR in a notable way, but the models were not able to reach a consistent accuracy at higher SNRs and therefore required more training data to be able to become a more reliable model.

**Figure 11:**
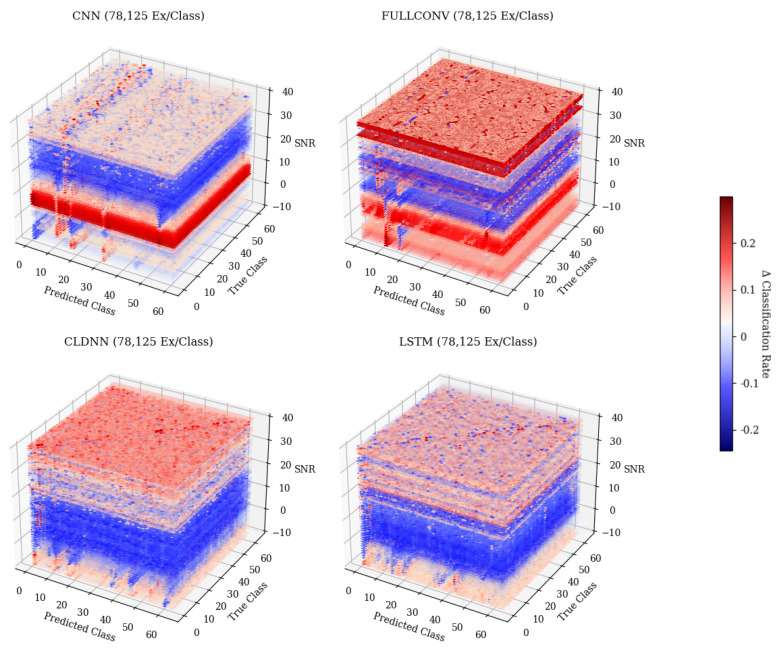
Three-dimensional gradient representation depicting the rate of change of accuracies across SNRs for each of the individual model architectures trained with 78,125 ex/class. Darker colors indicate a negative rate of change, while lighter colors indicate a positive rate of change.

Figure 11 shows a 3-D representation of the gradients taken across SNRs for each of the models trained with 78,125 examples per class. These figures show the patterns with which the rate of change of classifications changed for each True and Predicted Classes across SNsR. An examination of this approach showed that each of the models, at each training data quantity, generally showed the same trends discovered when examining the overall change in accuracy depicted in Figure 10. It is worth noting, however, that these 3-D representations depict the rate of change of classification rates across specific emitters that were commonly misclassified, which the representation in Figure 10 cannot do. Across SNRs, it can be seen that the general pattern depicted that at higher SNR values, the rate of change approached 0.0 for each model, but when examining the commonly misclassified emitters, it can be seen that this pattern differed. Commonly misclassified emitters tended to produce a high rate of change in classification rates across all SNR values, implying that as SNR increased, the amount that each of these emitters classified increased as well. This further supported the belief that error patterns were emitter-dependent, as when there was a higher SNR, the signals from emitters were more easily identified, ensuring that models learned features from signals rather than noise. An increase in the classification rate of specific emitters as signals learned from the emitters became more prevalent, implying that specific emitters generalized better to the features being learned and were therefore able to impact the classification results of the whole model.

### 4.3. Ensemble Analysis

In order to mitigate the weaknesses of each of the explored models that resulted in the discovered error patterns, this work designed an ensemble model that was capable of performing classifications with error patterns that were much more homogeneous than that of each of the individual models used to create the ensemble. An examination of the baseline accuracies, depicted in Figure 4 and Figure 5, shows that the ensemble models did not respond to SNR in the same manner as the weaker learners. Across SNRs, classification accuracy remained consistent, indicating that these ensemble models were robust to changes in the SNR. Across training data quantities, the ensemble models responded in the same manner as the weaker learners, with classification accuracies increasing as training data quantity increased. It is worth noting that at high SNR values, the classification accuracies of the CLDNN and LSTM models outperformed the ensemble; however, at low SNRs, the accuracies of the CLDNN and LSTM models harshly diminished, while the ensemble remained constant. This introduced a trade-off when using the ensemble model, where accuracy at high SNRs was lost in exchange for consistency across all possible SNR values. This trade-off motivates the use of this model in deployed use case scenarios where the SNR of collected data cannot be guaranteed; using the ensemble ensures the best possible overall performance without prior knowledge of SNRs.

**Figure 12:**
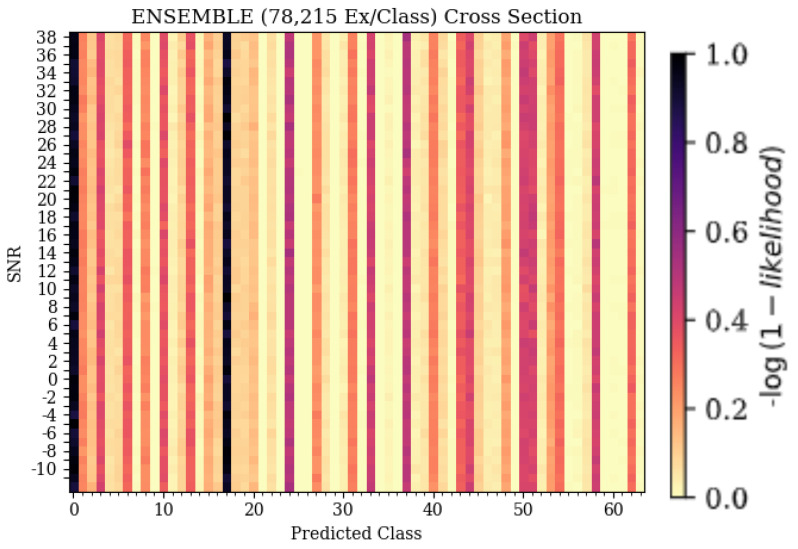
Cross sections taken from the ensemble model’s 3-D confusion matrix depiction, showing Predicted Class on the X-axis and SNR on the Y-axis. The dark colors signify the emitters that the models were consistently predicting when the true emitter was emitter 0.

The 3-D confusion matrix depictions created for the ensemble models can be seen in Figure 8. At each training data quantity, it can be seen that regardless of SNR, classification accuracy and error patterns remained consistent, whereas for each of the weaker learners, error patterns changed with SNR. At the lowest training data quantity, it can be seen that the ensemble model suffered from some patterns in misclassification, having some parallel patterns that indicated common misclassification as a specific subset of emitters that matched the same subset previously discussed, being emitters 11, 17, and 54. This pattern further supported the conclusion that these errors were emitter-dependent rather than architecture-dependent, as the ensemble would not produce consistent errors unless they were present in each model used to create the ensemble. As the training quantity increased, error patterns became evenly distributed across the feature space, leaving only the desired diagonal pattern that indicated accurate classifications. This even distribution of errors implied that classification failures of the ensemble model were suitably more random than the weaker learners that comprised it. Further, examining a cross-section of the ensemble taken across Predicted Class and SNR, shown in Figure 12, showed many classification decisions, implying that errors were fairly random. A random error pattern implies that attackers will not be able to exploit emitter-dependent patterns and, therefore, will have an equal chance of being incorrectly identified regardless of approach, meaning that the designed model is more robust to potential attacks.

**Figure 13:**
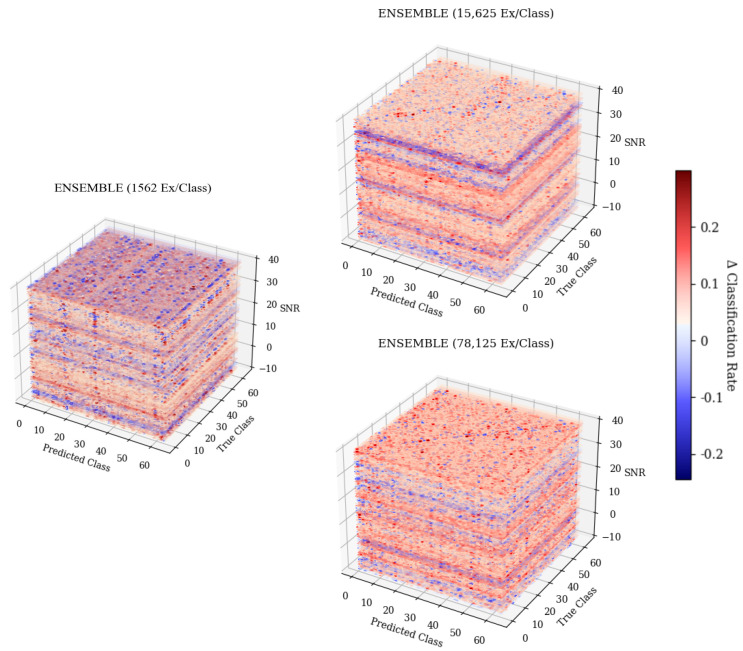
A 3-D gradient representation depicting the rate of change of accuracies across SNRs for the ensemble architecture. Models were trained with 1562 ex/class, 15,625 ex/class, and 78,125 ex/class.

A study of the ensemble model’s accuracy gradients, shown in Figure 10, and classification rate gradients, shown in Figure 13, showed that the ensemble models produced minimal changes in both accuracy and classification rates across SNsR. This further showed that the designed ensemble architecture was resilient to changes in SNR and that the ensemble was more resilient to emitter-related errors than the other models studied, as error patterns were repeated less and, therefore, changes in classification were consistent.

At higher SNRs, the results of the LSTM model and the CLDNN model, whose architecture combined the strength of convolutional layers and LSTMs, outperformed the ensemble approach in raw accuracy when measured on individual examples, but the ensemble approach’s ability to maintain performance across all SNR values as well as more uniformly distribute errors in classification decision makes it a more desirable model. Further, decisions from the ensemble model can be more effectively aggregated over multiple input examples to build stronger output decisions that rival those of the LSTM and CLDNN accuracies at high SNRs. This ensemble model excels over the other model architectures, as irregular classification error patterns and consistent accuracy across SNR values with only slightly lower aggregated accuracy than the CLDNN and LSTM at high SNRs make it the most effective model for mitigating error patterns and producing a model that is the most robust to potential attacks.

### 4.4. Entropy Analysis

**Figure 14:**
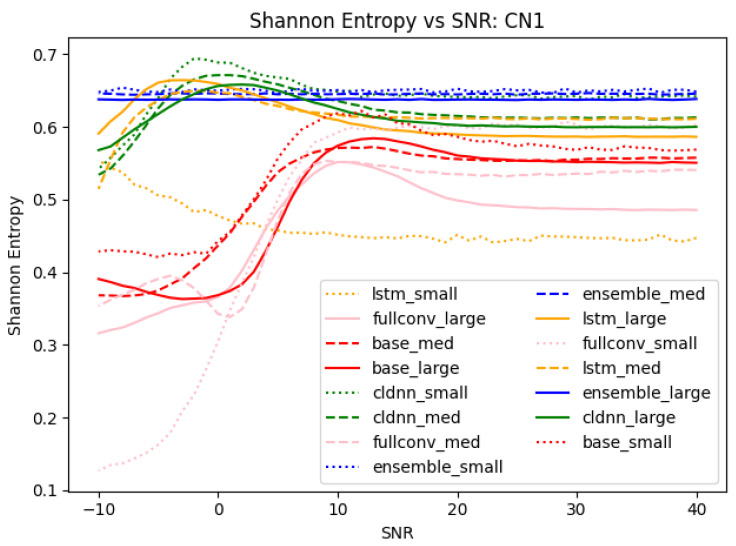
Shannon entropy for each of the models trained on data collected on CN1. Entropy values were normalized to the maximum entropy of a 64-class classification problem. In the legend, “small”, “med”, and “large” refer to training data quantities of 1562 ex/class, 15,625 ex/class, and 78,125 ex/class, respectively.

An analysis of Shannon entropy across SNRs was performed to support the analyses drawn from the 3-D confusion matrices in Section 4.2 and Section 4.3 that identified patterns in classification errors. The equation for Shannon entropy was as follows:

$$ShannonEntropy\left(bits\right)=-\sum_{i=1}^{n}p_{i}*\log_{2}p_{i}$$

where *n* is equal to the total number of classes [44]. To analyze the entropy of only mis-classifications, the diagonal matching true class to predicted class could be removed from each confusion matrix, thus making *n* = 63 for a 64-class problem. The maximum Shannon entropy of the mis-classifications could then be calculated to 5.977 bits using an evenly distributed probability distribution with *n* = 63. The calculated normalized Shannon entropy values for each model trained on data collected on CN1 and tested at SNR values between −10 and 40 can be seen in Figure 14.

A normalized Shannon entropy value of 1.0 indicated a perfectly random distribution of errors. Entropy values less than 1.0 indicated that the distribution of errors were skewed towards a smaller number of classes within the set of total available classes. An analysis of Figure 14 shows that at low SNRs, the base CNN, CLDNN, LSTM, and fullconv models each produced low entropies, indicating that error behaviors were biased and concentrated into a smaller subset of classes. As SNR increased, the entropy of these models increased, indicating that the distribution of classification errors became more randomly distributed but still did not show a perfectly random distribution. These results align with the findings in Section 4.2, showing that as SNR increased, the distribution of classification errors became more random. The Shannon entropy metric score provided a quantitative value indicating the presence of mis-classification patterns, while Figure 7 provides a simple visual to identify those patterns. An analysis of the ensemble results shown in Figure 14 showed that the distribution of error behaviors was biased towards certain classes; however, this bias remained consistent across SNR values. Stated differently, even at high SNRs, all of the models considered, even the composite ensemble model, made mistakes in somewhat predictable patterns. These findings align with the analyses in Section 4.3 stating that the designed ensemble model performs much more robustly in the presence of SNR variations. A qualitative metric showing consistent error behavior across SNRs further supported the ensemble model being the most effective model for mitigating error patterns in varying conditions. Utilizing the Shannon entropy metric in combination with Figure 8 provides both a quantitative metric that identifies the presence of mis-classification errors in the model and a visual representation of the errors to easily identify them.

## 5. Conclusions and Future Work

This work presented a novel approach for analyzing the failure patterns of different SEI model architectures using 3-D confusion matrices and proposed an ensemble model architecture designed to mitigate the discovered weaknesses. Four selected architectures were examined across a range of SNR values and training data quantities to isolate failure patterns that may be specific to one particular architecture or that are a necessary consideration for SEI as a whole. Further, a fifth architecture, an ensemble method, was designed to combine the learned features of each of the four other architectures in an effort to randomize the error patterns of the trained model.

**Figure 15:**
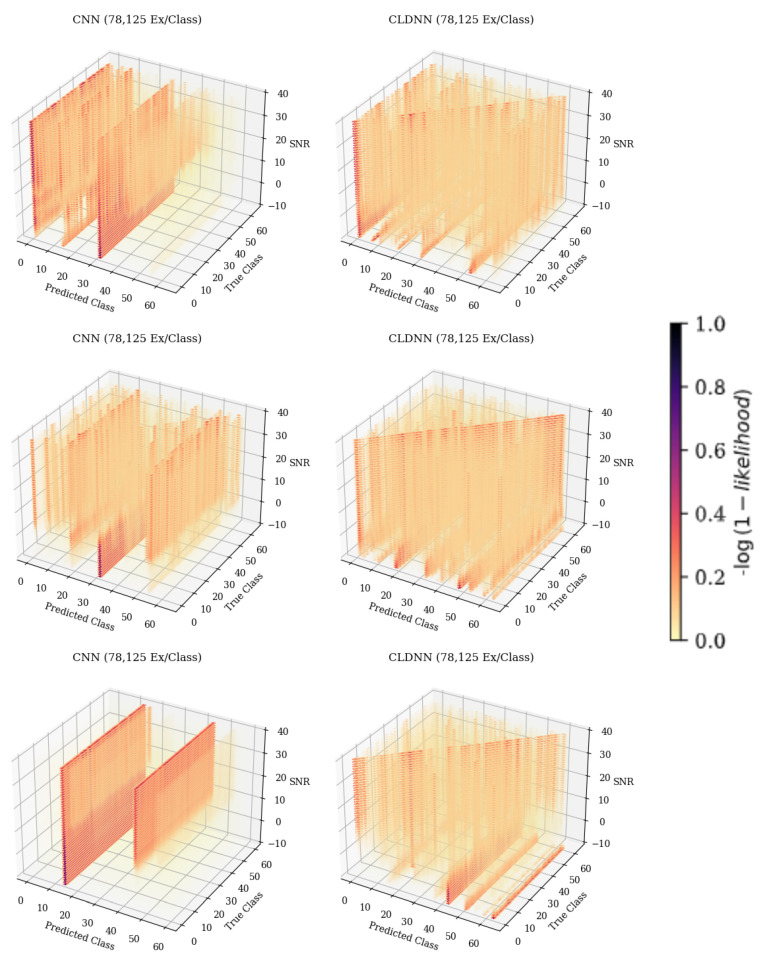

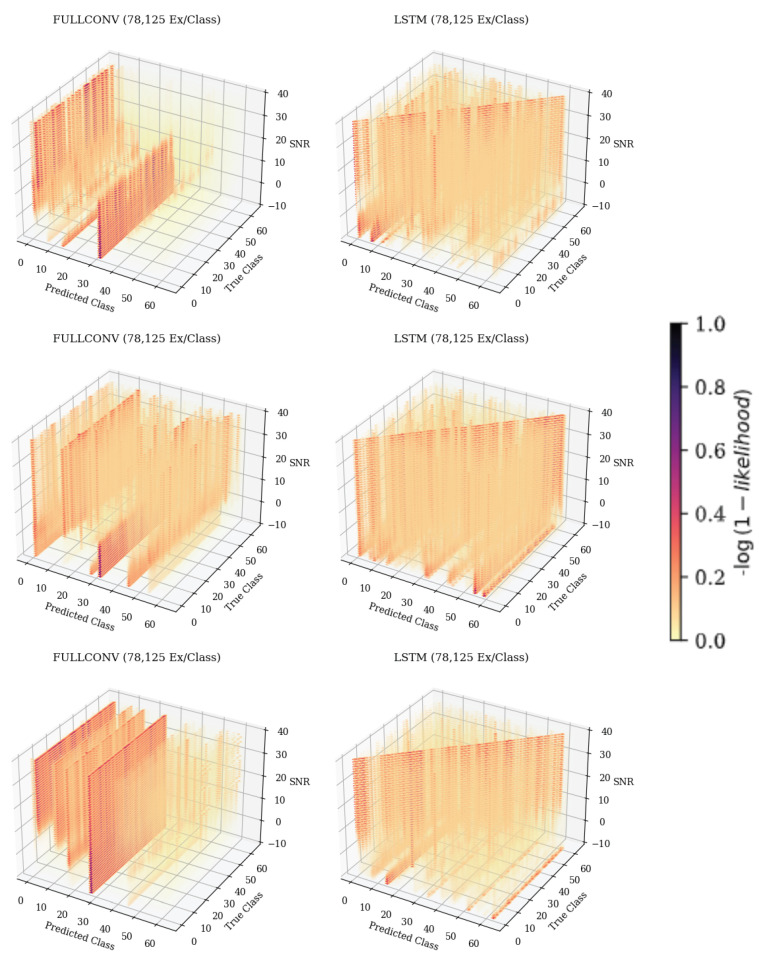
The 3-D confusion matrices from data collected on CN2 (**top**), CN3 (**middle**), and CN4 (**bottom**), depicting accuracies across SNRs for each of the individual model architectures trained with 78,125 ex/class. The darker the color, the more common the classification.

The results show that the 3-D analysis proves effective at providing a fast and efficient way to visualize the classification error patterns of a trained model across a variable of interest. At lower training data quantities, models produced significant misclassification patterns that were consistent across architectures. These consistent error patterns indicated that model failures were emitter-dependent, which was quantitatively supported using the Shannon entropy metric. At higher training data quantities, the desired classification pattern appeared; however, incorrect classifications patterns could still be seen, implying that an increase in training data quantity did not remove emitter-dependent error patterns. Additional confusion matrices showing recurring error patterns for data collected from CN2, CN3, and CN4 can be seen in Figure 15. These recurring patterns and Shannon entropy values indicated that errors were not homogeneously distributed, which appeared to be a common weakness in the RFML-based SEI algorithms caused by the features that the models were learning. Knowledge of this bias might in the future be combined with observed SNR values to refine second-choice classifications that reflect commonalities in a learned feature set and potentially even begin to characterize those features.

In order to mitigate the lack of randomization in erroneous classifications and create a model that does not suffer from the weaknesses of the four models explored in this work, an ensemble architecture was created that combined the learned feature spaces of each. This ensemble produced a classification accuracy that was consistent across SNR values and increased as training data quantity increased. When a multinomial aggregation technique was applied to the ensemble model, emulating the collection of data over a one-second span, the classification accuracies of the ensemble surpassed or rivaled that of each of the other architectures explored while remaining consistent across SNRs. Further, the 3-D confusion matrices of the ensemble model showed fewer error patterns at higher training data quantities, which was supported by Shannon entropy scores and implied that classification errors were more random. This means that the ensemble method is the most immune to attack threats of the models explored in this work. The designed ensemble architecture presents an approach that stabilizes accuracy across varying SNR conditions. This approach produces less frequently repeated classification errors and consistent classification accuracy results that rival or outperform the other models studied regardless of SNR. Given that this model achieved high performance on a large dimensionality SEI problem using 64 different emitters, the ensemble presents a strong, viable approach for performing SEI with as few weaknesses as possible.

In order to further explore the error patterns created by these model architectures, future work can be conducted to reverse engineer the features learned by each model. An understanding of which features are the cause of recurring misclassifications can help model designers to create SEI models that produce more uniformly distributed error patterns without requiring the use of the computational power and time required to train an ensemble method.

Additionally, the relevant range of SNRs present within the datasets used to train a model can be varied and model results studied in a manner similar to [45,46] to further strengthen the understanding of RFML algorithm performance. One approach to doing so could be to train several models using varying datasets: one collected at a fixed SNR value, another collected at small variations in SNR surrounding the previous fixed value, and a third collected using large SNR variations spanning the entirety of a relevant SNR range. The models trained using these datasets, in combination with the analysis approaches presented in this work, could provide detailed 3-D figures that allow for precise conclusions and comparisons to be drawn as to the impacts of SNR variation on SEI performance. Further, an ensemble technique similar to that designed in this work could be applied to the trained models to explore how each SNR range affects algorithm robustness when applied to the entire space.

Further, the insights gained from this study can be applied to the transient analysis of signal features. The transient portions of a signal have been shown to include a substantial quantity of radio frequency genuine features required for training accurate classification models [47]. The use of the transient portion of a signal also allows for accurate models to be created from smaller datasets [2] as the larger bulk portion of a signal is no longer required. Further study comparing the models examined in this work trained on data containing only transients versus only bulk data can provide valuable insights as to the robustness of these models by isolating their error patterns in various conditions.

Additional areas of interest include evaluating how the trained models break outside of their trained ranges, how models perform on data collected in non-line-of-sight propagation scenarios [25], and the impacts of the hardware implementations [48]; taken collectively, these explorations of how RFML-based SEI algorithms break are expected to improve the real-world deployability of the resulting design.

## Figures and Tables

**Table 1:** Summary of model architectures used in this work.

Model Architecture	Structure	Training Time	Reference
CNN	2-D Convolutional and	1562 ex/class: 40 min 15,625 ex/class: 6.54 h 78,125 ex/class: 32.2 h	[41]
	Fully Connected Layers		
	with ReLU Activation and		
	Batch Normalization		
FullConv	Modifed CNN structure	1562 ex/class: 40 min	[42]
	with Various Sizes of	15,625 ex/class: 6.48 h	
	Convolutional Layers	78,125 ex/class: 32.3 h	
LSTM	1-D CNN and Recurrent	1562 ex/class: 41 min	[43]
	Connections as Recurrent	15,625 ex/class: 6.65 h	
	Neural Network (RNN)	78,125 ex/class: 32.8 h	
CLDNN	1-D Convolutional and	1562 ex/class: 41.5 min	[10]
	LSTM Layers with ReLU	15,625 ex/class: 6.58 h	
	Activation and	78,125 ex/class: 33.0 h	
	Batch Normalization		
Ensemble	Combines CNN, FullConv,	1562 ex/class: 43 min	[10,41]
	LSTM, and CLDNN	15,625 ex/class: 7.14 h	
	through Bagging	78,125 ex/class: 34.8 h	

## Data Availability

The data presented in this study are available upon request to the corresponding author due to size constraints. The datasets presented in this article will be considered for separate publication after sponsor review. Requests to access the datasets should be directed to the corresponding author.
